# Supplementary material for: Mefloquine, a Potent Anti-severe Acute Respiratory Syndrome-Related Coronavirus 2 (SARS-CoV-2) Drug as an Entry Inhibitor in vitro
Source: Front Microbiol. 2021 Apr 30;12:651403. doi: 10.3389/fmicb.2021.651403 (PMC8119653; doi:10.3389/fmicb.2021.651403)
Supplement: Supplementary file 1 [file Data_Sheet_1.docx]

# Supplementary Notes

# Quantification of the drug dose-response curves.

# The typical dose-response curves of a single antiviral drug can be analyzed using the following Hill function (Koizumi et al. 2017) (Fig. 2B):

#

$$\boldsymbol{f}_{\boldsymbol{u}}\boldsymbol{=}\frac{\boldsymbol{1}}{\boldsymbol{1+}\left( \frac{\boldsymbol{D}}{\boldsymbol{IC}_{\boldsymbol{50}}} \right)^{\boldsymbol{m}}}\boldsymbol{.}\left( \boldsymbol{1} \right)$$

# Here, $\boldsymbol{f}_{\boldsymbol{u}}$ represents the fraction of infection events unaffected by the drug (i.e., $\boldsymbol{1-f}_{\boldsymbol{u}}$ equals the fraction of drug-affected events). $\boldsymbol{D}$ is the drug concentration, $\boldsymbol{IC}_{\boldsymbol{50}}$ is the drug concentration that achieves 50% inhibition of activity, and $\boldsymbol{m}$ is the slope of the dose-response curve (i.e., Hill coefficient) (Koizumi et al. 2017). Dose-response curves for drugs with higher $\boldsymbol{m}$ values show stronger antiviral activity at the same normalized drug concentration when the drug concentration is higher than the $\boldsymbol{IC}_{\boldsymbol{50}}$ (Fig. 2B). Least-square regression approach was used to fit Eq. (1) to dose-response data and estimate the values of $\boldsymbol{IC}_{\boldsymbol{50}}$ and $\boldsymbol{m}$. Those estimated values for each drug against SARS-CoV-2 are summarized in Table S1.

# Expected anti-SARS-CoV-2 effect of double-drug combinations by Bliss independence.

# We evaluated the effect of double-drug combinations for Bliss independence, widely used to analyze drug combination data (Bliss and Fisher 1953; Kobayashi et al. 2014; Koizumi and Iwami 2014; Tallarida 2001). Bliss independence assumes that each drug acts on different targets, and is defined as:

#

$$\boldsymbol{f}_{\boldsymbol{u}}^{\mathbf{Bcom}}\boldsymbol{=}\boldsymbol{f}_{\boldsymbol{u}}^{\boldsymbol{A}}\left( \boldsymbol{D} \right)\boldsymbol{\times}\boldsymbol{f}_{\boldsymbol{u}}^{\boldsymbol{B}}\left( \boldsymbol{D} \right)\boldsymbol{,}\mathbf{(2)}$$

# where $\boldsymbol{f}_{\boldsymbol{u}}^{\mathbf{Bcom}}$, $\boldsymbol{f}_{\boldsymbol{u}}^{\boldsymbol{A}}$ and $\boldsymbol{f}_{\boldsymbol{u}}^{\boldsymbol{B}}$ are the fractions of infection events unaffected by the combined drugs A (i.e., Nelfinavir: NFV) and B (i.e., Mefloquine: MFQ) expected by the Bliss model, single drug A and single drug B defined by Eq. (1), respectively. Using Eq. (2), we expected the anti-SARS-CoV2 effects of combined drugs A and B, $\boldsymbol{1-}\boldsymbol{f}_{\boldsymbol{u}}^{\mathbf{Bcom}}$, from the anti-SARS-CoV-2 effects of the single drugs.

# Determination of synergism.

# To determine the synergy between NFV and MFQ, both $\boldsymbol{f}_{\boldsymbol{u}}^{\mathbf{Bcom}}$ in Eq. (2) and the mean value of NFV and MFQ combination treatment data (e.g., $\boldsymbol{f}_{\boldsymbol{u}}^{\mathbf{EXcom}}$) were used to calculate $\boldsymbol{f}_{\boldsymbol{u}}^{\mathbf{Bcom}}\boldsymbol{/}\boldsymbol{f}_{\boldsymbol{u}}^{\mathbf{Excom}}$. If this value is equal to 1 (i.e., $\boldsymbol{f}_{\boldsymbol{u}}^{\mathbf{Bcom}}\boldsymbol{/}\boldsymbol{f}_{\boldsymbol{u}}^{\mathbf{Excom}}\boldsymbol{=1}$), then synergy is not observed (the white region in Fig. 4B). If $\boldsymbol{f}_{\boldsymbol{u}}^{\mathbf{Bcom}}\boldsymbol{/}\boldsymbol{f}_{\boldsymbol{u}}^{\mathbf{Excom}}\boldsymbol{>1}$, then $\boldsymbol{0<}\boldsymbol{f}_{\boldsymbol{u}}^{\mathbf{Excom}}\boldsymbol{<}\boldsymbol{f}_{\boldsymbol{u}}^{\mathbf{Bcom}}\boldsymbol{<1}$, indicating stronger antiviral activity in double combination treatment than expected by Bliss model, suggesting synergy (the orange region in Fig. 4B).

# Prediction of the antiviral effect of MFQ and HCQ in clinical settings.

# We used the following simple mathematical model which was also employed in (Ohashi et al. 2021):

#

$$\frac{\boldsymbol{df(t)}}{\boldsymbol{dt}}\boldsymbol{=-}\left( \boldsymbol{1-\eta}\left( \boldsymbol{t} \right)\boldsymbol{\times H}\left( \boldsymbol{t} \right) \right)\boldsymbol{\beta f}\left( \boldsymbol{t} \right)\boldsymbol{V}\left( \boldsymbol{t} \right)\boldsymbol{,}\left( \boldsymbol{1} \right)$$

#

$$\frac{\boldsymbol{dV(t)}}{\boldsymbol{dt}}\boldsymbol{=}\left( \boldsymbol{1-\eta}\left( \boldsymbol{t} \right)\boldsymbol{\times H}\left( \boldsymbol{t} \right) \right)\boldsymbol{\gamma f}\left( \boldsymbol{t} \right)\boldsymbol{V}\left( \boldsymbol{t} \right)\boldsymbol{-\delta V}\left( \boldsymbol{t} \right)\boldsymbol{,}\left( \boldsymbol{2} \right)$$

# where $\boldsymbol{f(t)}$ and $\boldsymbol{V(t)}$ are the ratio of uninfected target cells and the amount of virus, respectively. The parameters $\boldsymbol{\beta, \gamma}$, and $\boldsymbol{\delta}$ represent the rate constant for virus infection, the maximum rate constant for viral replication and the death rate of infected cells, respectively. $\boldsymbol{H}\left( \boldsymbol{t} \right)$ is a Heaviside step function defined as $\boldsymbol{H}\left( \boldsymbol{t} \right)\boldsymbol{=0}$ if $\boldsymbol{t<T}$: otherwise $\boldsymbol{H}\left( \boldsymbol{t} \right)\boldsymbol{=1}$, where $\boldsymbol{T}$ is the initiation timing of the treatment, and the anti-SARS-CoV2 effect of MFQ for $\boldsymbol{t>T}$ are described as follows:

#

$$\boldsymbol{\eta}\left( \boldsymbol{t} \right)\boldsymbol{=}\boldsymbol{1-f}_{\boldsymbol{u}}\left( \boldsymbol{D}\left( \boldsymbol{t} \right) \right)\boldsymbol{=1-}\frac{\boldsymbol{1}}{\boldsymbol{1+}\left( \frac{\boldsymbol{D}\left( \boldsymbol{t} \right)}{\boldsymbol{I}\boldsymbol{C}_{\boldsymbol{50}}} \right)^{\boldsymbol{m}}}\mathbf{,}\left( \boldsymbol{3} \right)$$

#

$$\boldsymbol{D}\left( \boldsymbol{t} \right)\boldsymbol{=}\boldsymbol{C}_{\boldsymbol{max}}\boldsymbol{e}^{\boldsymbol{-kt}}\boldsymbol{,}\left( \boldsymbol{4} \right)$$

# where $\boldsymbol{C}_{\boldsymbol{max}}$ and $\boldsymbol{k}$ are the peak drug concentration and the elimination rate for the corresponding drug, respectively. We used the same values of $\boldsymbol{\beta, \gamma,\delta}$, and $\boldsymbol{V}\left( \boldsymbol{0} \right)$ as previously defined (Ohashi et al. 2021), and the values of parameters in Eq. (3) and (4) for MFQ and HCQ are summarized in Table S1 and S2. The MFQ and HCQ antiviral activity was calculated with the expected pharmacokinetics in the human lung, based on the pharmacokinetics information for human peripheral blood and distribution to the lung (Desjardins et al. 1979; Jones et al. 1994; Chhonker et al. 2018) (see Table S2).

# Supplementary Figure

#
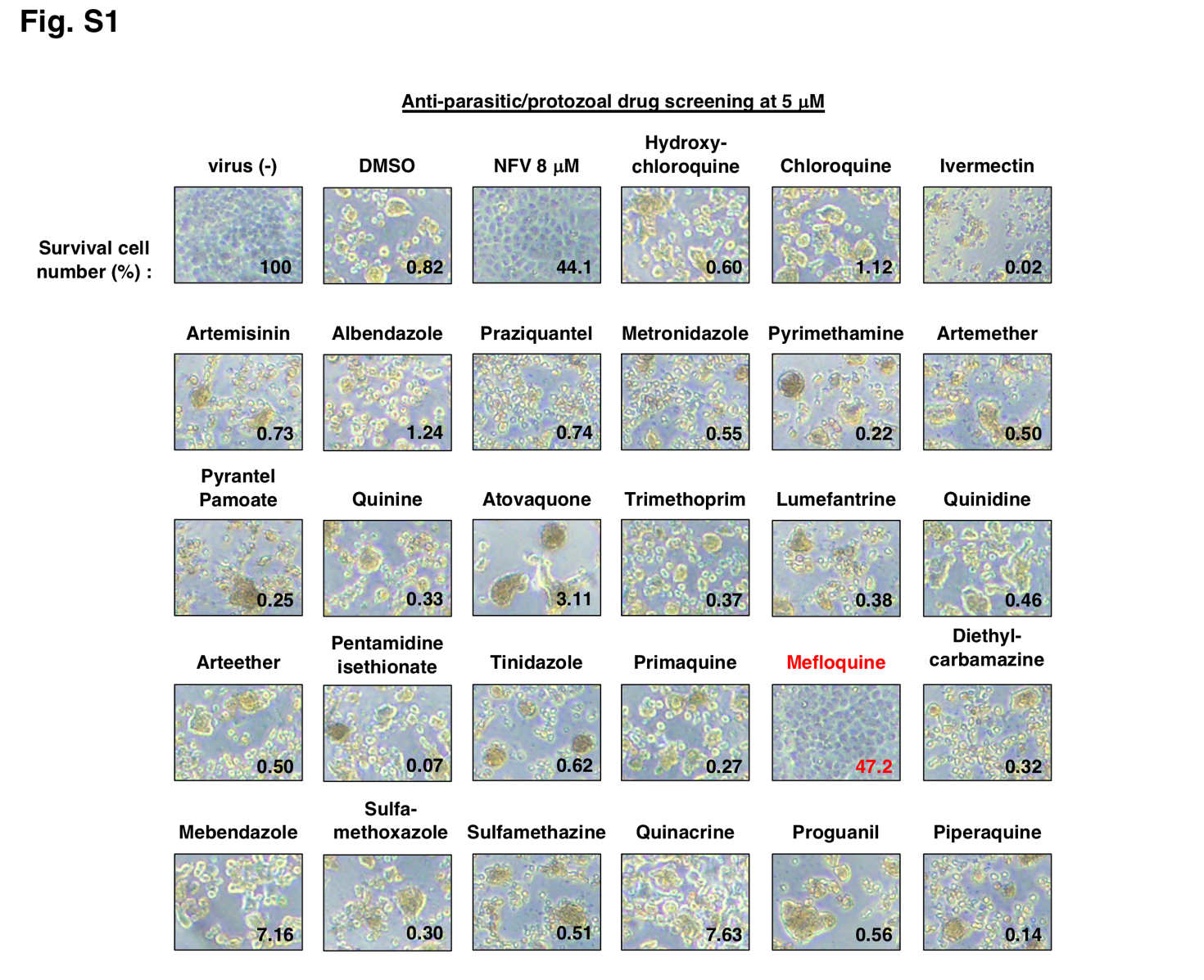


# Figure. S1. Screening of approved anti-parasitic/anti-protozoal drugs by the cytopathic effect assay of SARS-CoV-2. VeroE6/TMPRSS2 cells were treated with 5 μM of the indicated compounds, as shown in Fig. 1A. The figures show the cells observed by microscopy. The cell numbers were quantified with a high content imaging analyzer and are shown as the percentage of the uninfected sample [virus (-)] in the bottom of each image. NFV, suggested to inhibit SARS-CoV-2 infection, was used as positive control.

#
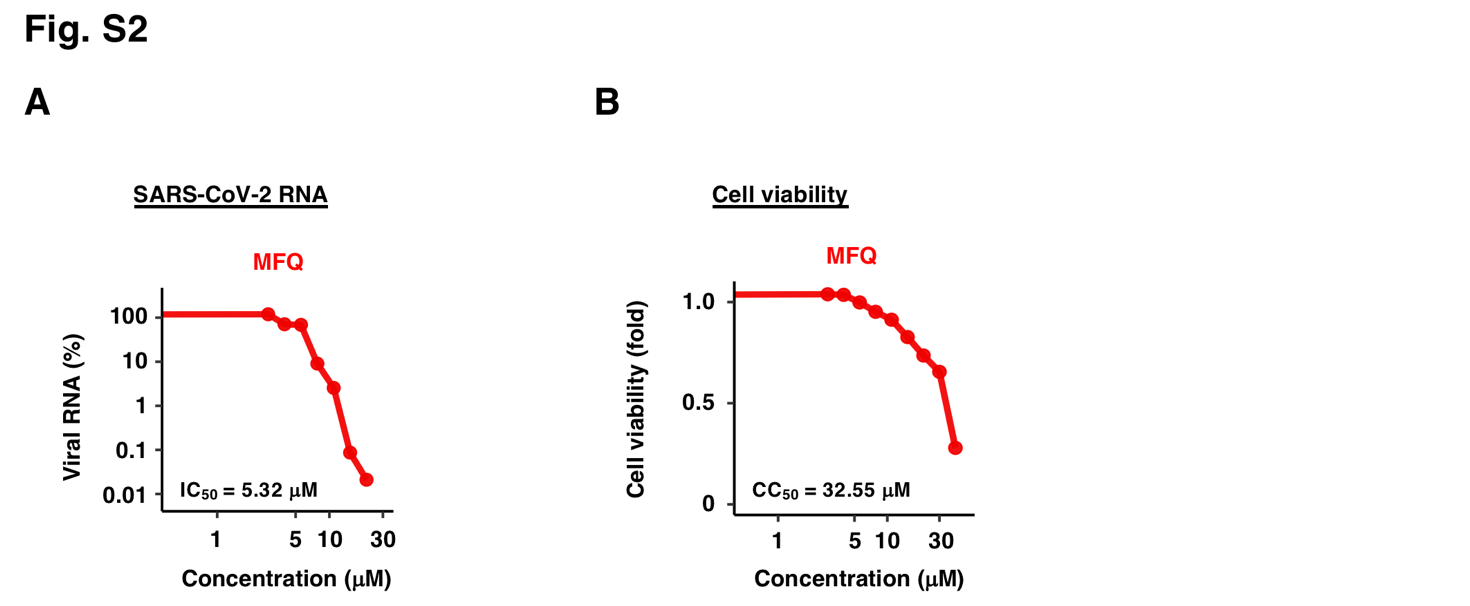


# Figure. S2. Antiviral and cytotoxic profile of MFQ in human lung epithelial-derived Calu3 cells. MFQ was treated at varying concentrations. (A) Antiviral activity was determined by quantifying the viral RNA in the supernatant 72 h post-infection. Calculated IC_50_ value of MFQ is 5.32 μM. (B) Cell viability was measured by counting the number of survival cells stained with DAPI. CC_50_ was calculated as 32.55 μM.

# Supplementary Tables

# Table S1. Estimated characteristic parameters of the tested antiviral drugs

| Drug (unit) | Class | ${IC}_{50}$ | $m$ |
| --- | --- | --- | --- |
| Single-drug treatment | | | |
| Hydroxychloroquine (µM) | EI | 1.937 | 1.555 |
| Primaquine (µM) | unknown | 2.473 | 0.571 |
| Mefloquine (µM) | EI | 1.285 | 3.738 |
| Quinine (µM) | unknown | 14.94 | 0.573 |
| Quinidine (µM) | unknown | 3.676 | 0.564 |
| Combination treatment | | | |
| Nelfinavir (µM) | RI | 2.323 | 15.56 |
| Mefloquine (µM) | EI | 0.907 | 2.383 |

# RI, replication inhibitor; EI, entry inhibitor

# $\boldsymbol{I}\boldsymbol{C}_{\boldsymbol{50}}$, 50% inhibitory concentration

# $\boldsymbol{m}$, the slope of the dose-response curve (i.e., Hill coefficient)

# Table S2. Summary of pharmacokinetic parameters of MFQ 1,000mg and HCQ 400 mg dose

| Parameter name | Symbol | Unit | Value* | |
| --- | --- | --- | --- | --- |
|  |  |  | MFQ | HCQ |
| Maximum concentration | $C_{max}$ | µM | 21.6 | 35.4 |
| Degradation rate | $k$ | day^-1^ | 0.0395 | 1.16 |

# *Expected pharmacokinetics information in a human lung. We estimated the scaling parameter of $\boldsymbol{C}_{\boldsymbol{max}}$ between the lung and peripheral blood, from the distribution of MFQ in the lung (Jones et al. 1994) and the distribution rate of HCQ between the lung and peripheral blood in human assuming that of the same value reported in mice (Chhonker et al. 2018). We then calculated $\boldsymbol{C}_{\boldsymbol{max}}$ in the human lung multiplying that in the human peripheral blood by the scaling parameter. Since the half-life of MFQ and HCQ in the lung is not available, we assumed a degradation rate of MFQ in the lung of the same value reported in the plasma, and for HCQ, employed the half-life estimated from mouse lung (Chhonker et al. 2018).

**Supplementary References**

Bliss, C. I., and Fisher, R. A. (1953). Fitting the Negative Binomial Distribution to Biological Data - Note on the Efficient Fitting of the Negative Binomial. *Biometrics* 9 (2): 176-200. https://doi.org/Doi 10.2307/3001850.

Chhonker, Y. S., Sleightholm, R. L., Li, J., Oupický, D., and Murry, D. J. (2018). Simultaneous quantitation of hydroxychloroquine and its metabolites in mouse blood and tissues using LC-ESI-MS/MS: An application for pharmacokinetic studies. *J Chromatogr B Analyt Technol Biomed Life Sci* 1072: 320-327. https://doi.org/10.1016/j.jchromb.2017.11.026.

Desjardins, R. E., Pamplin, C. L., 3rd, von Bredow, J., Barry, K. G., and Canfield, C. J. (1979). Kinetics of a new antimalarial, mefloquine. *Clin Pharmacol Ther* 26 (3): 372-9. https://doi.org/10.1002/cpt1979263372.

Jones, R., Kunsman, G., Levine, B., Smith, M., and Stahl, C. (1994). Mefloquine distribution in postmortem cases. *Forensic Sci Int* 68 (1): 29-32. https://doi.org/10.1016/0379-0738(94)90376-x.

Kobayashi, T., Koizumi, Y., Takeuchi, J. S., Misawa, N., Kimura, Y., Morita, S., et al. (2014). Quantification of deaminase activity-dependent and -independent restriction of HIV-1 replication mediated by APOBEC3F and APOBEC3G through experimental-mathematical investigation. *J Virol* 88 (10): 5881-7. https://doi.org/10.1128/jvi.00062-14.

Koizumi, Y., and Iwami, S. (2014). Mathematical modeling of multi-drugs therapy: a challenge for determining the optimal combinations of antiviral drugs. *Theor Biol Med Model* 11: 41. https://doi.org/10.1186/1742-4682-11-41.

Koizumi, Y., Ohashi, H., Nakajima, S., Tanaka, Y., Wakita, T., Perelson, A. S., et al. (2017). Quantifying antiviral activity optimizes drug combinations against hepatitis C virus infection. *Proc Natl Acad Sci U S A* 114 (8): 1922-1927. https://doi.org/10.1073/pnas.1610197114.

Ohashi, H., Watashi, K., Saso, W., Shionoya, K., Iwanami, S., Hirokawa, T., et al. (2021). Potential anti-COVID-19 agents, cepharanthine and nelfinavir, and their usage for combination treatment. *iScience* 24 (4): 102367. https://doi.org/10.1016/j.isci.2021.102367.

Tallarida, R. J. (2001). Drug synergism: its detection and applications. *J Pharmacol Exp Ther* 298 (3): 865-72.
